# Effect of Supplemental Cyanocobalamin on the Growth Performance and Hematological Indicators of the White Pekin Ducks from Hatch to Day 21

**DOI:** 10.3390/ani9090633

**Published:** 2019-08-30

**Authors:** Zaheer Ahmad, Ming Xie, Yongbao Wu, Shuisheng Hou

**Affiliations:** Institute of Animal Science, Chinese Academy of Agricultural Sciences, Beijing 100193, China

**Keywords:** vitamin B12, pekin ducks, weight gain, feed intake, feed conversion ratio, hematological indicator

## Abstract

**Simple Summary:**

Vitamin B12 plays a key role in the normal functioning of the brain and nervous system as well as creation and regulation of nucleic acids (DNA and RNA). Furthermore, vitamin B12 plays a significant role in fatty acid metabolism and energy generation. Deficiency of vitamin B12 in animals may lead to weakness and anemia because it is involved in the formation of hemoglobin, which transports oxygen to body cells and red blood cells. Similarly, deficiency of vitamin B12 may also lead to hyperhomocysteinemia (increased level of homocysteine in the blood) which may depress immunity and cause cardiovascular diseases. There is no literature available regarding cyanocobalamin requirement for Pekin ducks. Therefore, the aim of our study is to determine its requirement. However, we find that cyanocobalamin has no influence on growth performance (weight gain), but it has more effect on hematological indicators (blood). On the basis of growth performance and hematological indicators we suggest that 0.02 mg cyanocobalamin/kg of feed is the dietary requirement of male Pekin ducks from hatch to day 21.

**Abstract:**

The experiment was conducted to evaluate the requirement of cyanocobalamin of male Pekin ducks from hatch to 21 days of age. A total of three-hundred-eighty-four, one-day-old meat-type male Pekin ducks were randomly allocated to six treatments, i.e., dietary cyanocobalamin (vitamin B12) concentrations of 0.00, 0.02, 0.04, 0.06, 0.08 and 1.00 mg/kg, respectively in their feed. Each treatment had eight replicated pens with eight ducks for each pen. Feed and water were provided ad libitum. The experiment was conducted for 21 days. Different growth parameters including average daily weight gain (ADG), average daily feed intake (ADFI), feed conversion ratio (FCR), and hematological indicators were evaluated because, on the basis of hematological indicators, the health and nutritional status of an animal can be accessed. It is observed that supplemental cyanocobalamin has no significant effect on ADG, ADFI, and FCR but it improves hematological parameters such as white blood cells, red blood cells, and its indices and platelet counts compared to the control group (*p* < 0.05). On the basis of growth performance and hematological indicators it is concluded that 0.02 mg cyanocobalamin/kg of feed is the dietary requirement of male Pekin ducks from hatch to day 21 of age.

## 1. Introduction

Growth of ducks is faster than all other poultry species [1]. To bias the increased growth rate, these have been genetically selected like all the other poultry species [2]. Numerous studies have suggested that vitamin B12 is necessary for chick growth and egg hatchability [3,4,5,6,7]. Deficiency of vitamin B12 has a critical impact on growth and existence of rats. It is also reported that the addition of high levels of protein in a diet with a deficiency of vitamin B12 causes high mortality [8]. In another study, Dryden and Hartman (1971) observed a steady increase in growth rate with every increased unit of protein content that might not be associated with the breakdown of surplus nitrogen but with complications in the disposal of the carbon skeleton of certain or total amino acids of added protein contents in vitamin B12 deficient diets [9].

The natural vitamin contents of diets depend upon ingredients and compositions that can vary considerably between particular feedstuffs based on the season, region, and processing conditions which can induce natural variations in vitamin contents. Natural dietary contents rarely provide the intakes thought to fulfill the needs of birds to meet their normal requirements and to provide a margin of safety to meet extra metabolic demands imposed by stress and other factors [10]. Whitehead (2002) [11] demonstrated that diets supplemented with vitamins play a vital role for disease prevention and treatment. He also illustrated that biological functions are carried out by the involvement of vitamins that allow an animal to use energy and proteins for growth, health, maintenance, reproduction, and feed conversion [11]. Poultry intake B12 either by feed supplementation or ingesting feces. This vitamin plays a vital role in the nervous system and proper brain functioning, homocysteine metabolism, energy metabolism, normal blood functions, cell division, and in the immune system [12]. Similarly it is also anti-anemic in function [13]. Vitamin B12 acts as a co-factor for L-methylmalonyl-CoA mutase and methionine synthase. Methionine synthase increases the rate of homocysteine conversion to methionine which is further required for DNA and RNA synthesis while L-methylmalonyl-CoA mutase converts L-methylmalonyl-CoA to Succinyl-CoA which is a crucial biochemical reaction in fat and protein metabolism. Succinyl-CoA is also required for hemoglobin synthesis [14].

Ceca of the birds synthesize vitamin B12 by using cobalt, but its production is lower than daily requirements, and suggestions were given to supplement diets with vitamin B12 [15]. In another study, researchers reported that for an increased growth rate and maximum feed utilization, a growing ration fortified with vitamin B12 had appreciable effects but it may not be optimum for achieving maximum feed efficiency [16]. Folic acid, vitamin B12, and dietary total non-structural carbohydrates have no effect on milk production and milk total solid yield. There was also a non-significant difference observed on average daily gains among the calves supplemented with these vitamins [17]. Vitamin B12 deficiency also caused health problems in many ways, from hematological manifestations to neurological disorders, varied as leucoenia, numbness in fingers, unsteadiness of gait, fasciculation, and macrocytic anemia [18]. Erythrocytes depend on vitamin B12 for their maturation and proliferation. Therefore vitamin B12 deficient erythrocytes cannot mature, resulting in hemolysis and hyperbilirubinemia [19].

Blood parameters are indices of the internal environment of the living body and they also indicate the health status of ducks [20]. To improve animals’ productivity, it is important to understand their physiology and hematological characteristics. For the establishment of a diagnostic baseline for blood characteristics, hematological studies are usually undertaken on farm animals as routine management practices [21,22]. Hematological constituents serve as authentic tools for monitoring animal health because they usually reflect the physiological reactions of an animal to its external and internal environment [23,24]. Hematological indicators are very important as they designate an animal’s health and nutritional status [25]. These parameters in poultry are also sensitive to stress reactions [26]. Hematological indicators are considered to be biomarkers for the immune system [27]. There is a limited literature available for hemogram parameters in vitamin B12 deficiency [28]. That is why we investigate these hematological indicators on different levels of vitamin B12 in Pekin ducks. The National Research Council (NRC) (1994) also has no data regarding the vitamin B12 requirement of meat-type Pekin ducks for growth and hematological indicators. Therefore, one of the main goals of this study is to estimate the requirement of cyanocobalamin as a supplement for the growth performance and hematology of male Pekin ducks from hatch to 21 days of age. 

## 2. Materials and Methods Estimate

### 2.1. Study Design

All procedures of this study were permitted by the Animal Care and Welfare Committee of the Institute of Animal Science, Chinese Academy of Agricultural Sciences (No. IASCAAS-AE-03, No. 20180416). A dose-response experiment with six supplemental vitamin B12 levels (0, 0.02, 0.04, 0.06, 0.08 and 1.00 mg/kg) was designed. Vitamin B12 deficient basal diet was prepared (Table 1) and it was comprised of 0.00 mg/kg total vitamin B12, according to the data of feed ingredients of the NRC (1994).

### 2.2. Chicks and Diets

The basal diet was formulated first and then six experimental diets were added with various supplemental levels of crystalline vitamin B12. A total of 384 one-day-old male White Pekin ducks with average body weights around 54 g were divided into 6 experimental groups and each group was replicated 8 times with 8 birds per pen. These ducklings were reared in steel cages with plastic floors (200 × 100 × 40 cm), from hatch to 21 days of age. They were raised and allocated randomly to an environmental control shed. The birds were offered feed and water ad libitum. Provision of water was done by using a drip-nipple, and feed was offered in pellet form. Birds were provided 24-h lighting, and temperature was maintained at 33 °C with relative humidity at 20% from 1 to 3 days of age, then temperature was decreased steadily to room temperature while relative humidity gradually increased to 65% until birds were 21 days of age.

### 2.3. Growth Performance and Hematological Parameters 

On the 21st day, the average daily weight gain, average daily feed intake, and feed conversion ratio (FCR) of ducks from every pen were calculated using the following formula.

$$\mathrm{Feed}\;\mathrm{conversion}\;\mathrm{ratio}\;\left(\mathrm{FCR}\right)\;=\;\frac{\mathrm{Feed}\;\mathrm{intake}}{\mathrm{Weight}\;\mathrm{gain}}$$

Feed intake and FCR were all corrected for mortality.

### 2.4. Sampling and Analysis

After fasting 12 h, two ducks from each pen were selected randomly for blood collection on the basis of average body weight gain of the corresponding pen. Three to four milliliters of blood were collected in an Ethylenediaminetetraacetic Acid (EDTA)-containing tube from the jugular vein of each selected bird. These blood samples were analyzed by using an automated hematological analyzer (ABX PENTRA DX 120, Munich, Germany).

### 2.5. Statistical Analysis

Data were analyzed using SPSS Statistics 22.0 (Statistical Packages for the Social Sciences, released December 2013, Armonk, New York, USA). The mean comparison test was applied by using Duncan’s new multiple-range test to find out the significant differences among the applied treatments at 5% the level of significance (*p* < 0.05).

## 3. Results

### 3.1. Growth Performance

The effect of dietary cobalamin on average daily weight gain (ADG), average daily feed intake (ADFI), and feed conversion ratio (FCR) of the experiment is described in Table 2. ADG, ADFI, and FCR showed non-significant results among the vitamin B12 supplemented groups and the control group (*p* > 0.05), as presented in Table 2.

### 3.2. Hematology

#### 3.2.1. White Blood Cells

On day 21, ducks from the control group had significantly lower (*p* < 0.05) values for the white blood cell count (WBC) and had significantly higher (*p* < 0.05) values for the percentage of intermediate cells (MID %) than the supplemented groups. However, there were no further significant differences among the vitamin B12 supplemented groups. The absolute value of granulocytes (GRA) and percentage of granulocytes (GRA %) were lower for the control (C) group as compared to the vitamin B12 supplemented groups, although these values were not statistically significant (*p* > 0.05). There were non-significant results observed for the absolute value of lymphocytes (LYM), percentage of lymphocytes (LYM %), and absolute value of intermediate cells (MID), as presented in Table 3.

#### 3.2.2. Red Blood Cells

At the end of the experiment, ducks with different levels of vitamin B12 had higher significant (*p* < 0.05) values for red blood cells (RBC), hemoglobin (Hb), hematocrit (HCT), mean corpuscular volume (MCV), mean corpuscular hemoglobin (MCH), and standard deviation of RBC distribution width (RDW-SD) compared to the control group (Table 4). By the end of the experiment, mean corpuscular hemoglobin concentration (MCHC) and coefficient of variation in RBC width (RDW-CV) values of the control ducks had increased more significantly (*p* < 0.05) than the treated ducks. The values of RBC, Hb, HCT, MCV, MCH, and RDW-SD of the control ducks were significantly lower (1.4 to 1.8 10^12^/L, 82.4 to 116.9 g/L, 0.17 to 0.26 L/L, 107.5 to 148.9 fL, 54.3 to 66.6 pg, and 43.2 to 63.9%) than supplemented ducks respectively. Results also concluded that vitamin B12 plays a vital role in the synthesis of blood necessary for the different proper and optimal types of functions for the ducks and their well-being (Table 4).

#### 3.2.3. Platelet Count

The values of the platelets indices by day 21 are shown in Table 5. The values of the platelet count, thrombocytocrit, and mean platelet volume of the control group had significantly lower values than the supplemented groups, (41.3 to 84.6 10^9^/L, 0.05 to 0.11 L/L and 11.4 to 13.7 fL) respectively, whereas the values of platelet distribution width of the control group were significantly higher (51.9 to 22.9%) than treated groups (Table 5).

## 4. Discussion

The influence of different levels of vitamin B12 on growth performance and hematological analysis was studied in Pekin ducks during the starter phase (0–21 days). There is no data available on the requirement of vitamin B12 in the Pekin duck, also the literature regarding vitamin B12 and Pekin ducks is limited. 

In our study there were no significant differences found for the average daily feed intake and average daily weight gain in the group without vitamin B12 supplementation and groups supplemented with vitamin B12 in the Pekin ducks, respectively. The mean ADG, ADFI, and FCR in the present study is similar to that described in ducks earlier with vitamin B12 [12]. Our results also match the findings of other researchers who found that increasing levels of vitamin E in the diet had no significant effect on live body weight gain, body weight gain, feed intake, and FCR [29].

Without supplementation of vitamin B12 in the feed, the ducks did not show any clinical signs related to vitamin deficiency because the liver can preserve vitamin B12 for long periods of time, even feeding a vitamin B12 deficient diet. They further illustrated that about 2–5 months may be required for the elimination of B12 preserved by hens to such an extent that progeny will hatch with a low reserve of vitamin B12 [30].

Vitamin B12 had no effect (Table 2) on growth performance (*p* > 0.05). This might be due to the short period of the experiment (21 days) and its very low requirement for proper growth. In the present study, ducklings hatched from parent flocks which were feeding according to the nutrient requirements of breeder ducks. Therefore, it can be assumed that the storage of vitamin B12 in the egg yolk was stabilized, and the freshly hatched ducklings had an optimum B12 depot in the liver and in the last part of the yolk sac, and that the liver maintained the level of vitamin B12 for 21 days in the ducklings even without its supplementation in the diet [12].

Supplementation of vitamin B12 had no significant effect on the production and fertility of eggs. It was also revealed that vitamin B12 deficiency in the breeder diet had adverse effect on growth of chicks and when the chicks were collected from breeders during hatching they were supplemented with vitamin B12 but their deficiency was fulfilled up to some extent. It is therefore concluded that the supplementation of vitamin B12 in the diet of hens was more effective than the chick diet for the growth in their early stage [7].

However, limited research work has been done on hematological profiles and limited information is available on these parameters in ducks. Vitamin B12 is essential for proper red blood cell production, neurological function, and DNA formation [31,32,33,34]. The effects of vitamin B12 on the hematological indicators are shown in (Table 3, Table 4 and Table 5). The hematological indicators weremeasured in WBCs, RBCs, and platelets after both the addition and subtraction of vitamin B12. Vitamin B12 supplemented groups showed significantly higher values than the control group (*p* < 0.05).

Vitamin B12 works as a co-factor for methionine synthase and L-methylmalonyl-CoA mutase. Methionine synthase speeds up the transformation of homocysteine to methionine. Methionine is mandatory for the creation of S-adenosylmethionine, which is a universal methyl donor for nearly 100 different substrates, including DNA, RNA, hormones, proteins, and lipids. L-methylmalonyl-CoA mutase converts L-methylmalonyl-CoA to Succinyl-CoA in the degradation of propionate, which is a critical biochemical reaction in fat and protein uptake. Succinyl-CoA is necessary for synthesis of hemoglobin [35].

Dietary supplementation with a combination of probiotics and prebiotics markedly increased the PCV, RBC, and Hb of guinea fowls [36]. Molasses also had significant effect on MCV and platelets in broiler chickens during hot dry seasons. Presence of high MCV may indicate an active erythropoiesis, as MCV is thought to be the average size of an individual erythrocyte [30]. Younger erythrocytes are larger than older ones [37,38]. Betaine had highly significant effects on hematological parameters in the meat-type ducks under stress conditions; specially values of RBC, HCT, HGB, MCV, MCHC, RDW, platelets PLT, PCT, and MPV were significantly higher (*p* < 0.05) in betaine added groups as compared to the control group but no further significant differences were found among the betaine supplemented groups [39]. Similarly the control group had significantly lower (*p* < 0.05) values for RBC, Hb, PCV, MCV, and MCH in different breeds of Omani goats from treated groups which were receiving dietary cobalt and cutaneous injections of hydroxycobalamin [40]. Hemorrhagic anemia and hemolytic anemia are caused by the decreased value of RBCs, whereas the decrease of hemoglobin causes microcythemia. It is also reported that hematocrit and packed red cells volume have an immense correlation with each other. Consequently, when the mean hematocrit decreases, the levels of hematoglobin and hemoglobin becomes lower [39]. In a study, MPV was found to be lower in patients with vitamin B12 deficiency than in the patients without vitamin B12 deficiency as a result of production of smaller platelets [18,41].

## 5. Conclusions 

To the best of our knowledge, this is the first time that someone determined the dietary requirement of cyanocobalamin for male Pekin ducks during the starter phase on the basis of growth performance and hematological indicators. However, we found that cyanocobalamin had a non-significant effect on growth performance, but it had more significant improvements on hematological indicators. On the basis of our findings we suggest that 0.02 mg cyanocobalamin/kg of the feed may be supplemented to improve hematological variables of Pekin ducks from hatch to day 21.

## Figures and Tables

**Table 1:** Composition of basal diet (% as fed).

Items	Content (%)
Ingredients	
Corn	63.6
Soyabean meal	32.4
Calcium hydrophashate	1.6
Lime stone	0.8
Premix ^1^	1
NACL	0.3
Methionine	0.2
Lysine	0.1
Calculated Composition	
Metabolizable energy (Mcal/kg) ^2^	2.9
Crude protein	20.03
Calcium	0.96
Nonphytic acid phosphor	0.42
Lysine	1.11
Methionine	0.51
Methionine + Cystine	0.85
Threonine	0.81
Tryptophan	0.25
Arganine	1.41
Valine	0.93
Isoleucine	0.81
Phosphorus	0.62
Vitamin B12	0

^1^ Supplied per kilogram of total diet: Cu (CuSO_4_·5H_2_O), 8 mg; Fe (FeSO_4_·7H_2_O), 60 mg; Zn (ZnO), 60 mg; Mn (MnSO_4_·H_2_O), 100 mg; Se (NaSeO_3_), 0.3 mg; I (KI), 0.4 mg; Mg(MgO), 200 mg; K(K_2_CO_3_), 1500; choline chloride, 1000 mg; vitamin A (retinyl acetate), 4000 IU; vitamin D_3_ (Cholcalciferol), 2000 IU; vitamin E (DL-α-tocopheryl acetate), 20 IU; vitamin K_3_ (menadione sodium bisulfate), 2 mg; thiamin (thiamin mononitrate), 2 mg; riboflavin, 10 mg; pyridoxine hydrochloride, 4mg; calcium-D-pantothenate, 20 mg; nicotinic acid, 50 mg; folic acid, 1 mg; and biotin, 0.20 mg.^2^ The values were calculated according to the apparent metabolizable energy of chickens.

**Table 2:** Effect of dietary vitamin B12 on average daily weight gain (ADG), average daily feed intake (ADFI), and feed conversion ratio (FCR) in meat-type Pekin ducks (mean ± SD).

Parameters	Vitamin B-12 (mg/Kg) ^1^						*p*-Value
	C	0.02	0.04	0.06	0.08	1	
ADG (g/bird/day)	55.73 ± 1.6	56.30 ± 0.9	56.72 ± 1.1	56.25 ± 1.5	56.35 ± 1.5	56.29 ± 1.6	0.853
ADFI (g/bird/day)	89.95 ± 4.7	92.10 ± 2.2	93.6 ± 2.8	92.4 ± 2.5	94.0 ± 3.4	94.1 ± 4.7	0.174
FCR	1.61 ± 0.10	1.63 ± 0.02	1.65 ± 0.02	1.64 ± 0.03	1.66 ± 0.04	1.67 ± 0.03	0.301

^1^ Dietary treatments where C = control (without vitamin B12), all others are vitamin B12 supplemented groups. Each mean represents values from 8 replicates (8 ducks/ replicate).

**Table 3:** Effect of dietary vitamin B12 on white blood cell count and its indices in meat-type Pekin ducks (mean ± SD).

Parameters	Vitamin B-12 (mg/Kg) ^1^						*p*-Value
	C	0.02	0.04	0.06	0.08	1	
WBC (10^9^/L)	126.7 ± 22.5 ^b^	139.2 ± 7.2 ^a^	141.8 ± 4.3 ^a^	137.3 ± 14.9 ^a^	142.5 ± 3.0 ^a^	133.8 ± 9.5 ^ab^	0.004
LYM (10^9^/L)	60.2 ± 13.4	62 ± 11.2	62.9 ± 7.2	64.1 ± 9.4	61.5 ± 8.3	60.9 ± 13.1	0.474
MID (10^9^/L)	20.4 ± 2.9	21.1 ± 3.2	20.9 ± 1	20.9 ± 1.8	20.7 ± 1.7 ^b^	20.5 ± 3.0	0.061
GRA (10^9^/L)	46.1 ± 33.9	61.1 ± 16.9	57.9 ± 8.7	54.2 ± 21.9	62.3 ± 9.9	54.5 ± 14.3	0.211
LYM (%)	51.7 ± 17.2	41.2 ± 9.1	44.6 ± 5.8	47.7 ± 10.4	41.9 ± 6	44.9 ± 9.6	0.139
MID (%)	16.5 ± 3.8 ^a^	15.2 ± 2.8 ^ab^	14.7 ± 0.7 ^b^	13.9 ± 1.2 ^b^	14.5 ± 1.1 ^b^	14.9 ± 2.4 ^b^	0.028
GRA (%)	33.3 ± 20.5	43.6 ± 11.4	40.7 ± 5.5	38.4 ± 11.3	43.6 ± 6.4	40.8 ± 10.9	0.161

^a,b^ Superscript letters show significant differences (*p* ≤ 0.05). ^1^ Dietary treatments where C = control (without vitamin B12), all other are vitamin B12 supplemented groups. Each mean represents values from 8 replicates (2 ducks/ replicate). WBC: White Blood Cells; LYM: Absolute value of lymphocytes; MID: The absolute value of the intermediate cell; GRA: Absolute value of granulocytes; LYM (%): Percentage of lymphocytes; MID (%): Percentage of intermediate cells; GRA (%): Percentage of granulocytes.

**Table 4:** Effect of dietary vitamin B12 on red blood cell count and its indices in meat-type Pekin ducks (mean ± SD).

Parameters	Vitamin B-12 (mg/Kg) ^1^						*p*-Value
	C	0.02	0.04	0.06	0.08	1	
RBC (10^12^/L)	1.4 ± 0.5 ^b^	1.7 ± 0.2 ^a^	1.6 ± 0.4 ^ab^	1.5 ± 0.4 ^b^	1.8 ± 0.1 ^a^	1.6 ± 0.3 ^a^	0.005
HGB (g/L)	82.4 ± 42.6 ^b^	111.9 ± 11.5 ^a^	103.1 ± 22.8 ^a^	109.1 ± 37.5 ^a^	111.8 ± 7.1 ^a^	116.9 ± 17.6 ^a^	0.001
HCT (L/L)	0.17 ± 0.11 ^b^	0.25 ± 0.03 ^a^	0.23 ± 0.05 ^a^	0.20 ± 0.09 ^b^	0.26 ± 0.02 ^a^	0.26 ± 0.04 ^a^	0.001
MCV (fL)	107.5 ± 38.6 ^b^	145.7 ± 3.5 ^a^	141.8 ± 5.3 ^a^	120.3 ± 28.6 ^b^	142.5 ± 3.5 ^a^	148.9 ± 2.9 ^a^	0.001
MCH (pg)	54.3 ± 12.15 ^b^	65.1 ± 4.6 ^a^	64.1 ± 6.67 ^a^	59.8 ± 12.12 ^b^	61.7 ± 4.05 ^a^	66.6 ± 4.3 ^a^	0.001
MCHC (g/L)	505.6 ± 82.3 ^a^	424.2 ± 22.1 ^b^	428.5 ± 33.9 ^a^	398.1 ± 57.6 ^b^	410.6 ± 20.9 ^b^	424.5 ± 21.6 ^b^	0.001
RDW-SD (fL)	43.2 ± 18.9 ^c^	64.4 ± 4.2 ^a^	63.4 ± 3.9 ^a^	52.6 ± 16 ^b^	61.4 ± 2.3 ^a^	63.9 ± 13.6 ^a^	0.001
RDW-CV (%)	32.1 ± 18.8 ^a^	13.7 ± 1.6 ^b^	15.1 ± 2.2 ^b^	27.9 ± 16.8 ^b^	15.2 ± 1.3 ^b^	13.1 ± 1.1 ^b^	0.001

^a,b,c^ Superscript letters show significant difference (*p* ≤ 0.05). ^1^ Dietary treatments where C = control (without vitamin B12), all others are vitamin B12 supplemented groups. Each mean represents values from 8 replicates (2 ducks/replicate).

**Table 5:** Effect of dietary vitamin B12 on platelet count and its indices in meat-type Pekin ducks (mean ± SD).

Parameters	Vitamin B-12 (mg/Kg) ^1^						*p*-Value
	C	0.02	0.04	0.06	0.08	1	
PLT (10^9^/L)	41.3 ± 23.4 ^c^	55.8 ± 33.9 ^ab^	76.7 ± 38.9 ^bc^	84.6 ± 33.7 ^a^	73.4 ± 23.1 ^bc^	43.6 ± 19.7 ^b^	0.001
PCT (L/L)	0.05 ± 0.03 ^b^	0.08 ± 0.05 ^ab^	0.11 ± 0.05 ^a^	0.11 ± 0.05 ^a^	0.09 ± 0.03 ^a^	0.06 ± 0.03 ^b^	0.001
MPV (fL)	11.4 ± 2.1 ^c^	13.4 ± 0.41 ^a^	13.7 ± 0.3 ^a^	12.4 ± 1.6 ^b^	13.5 ± 0.26 ^a^	13.5 ± 0.37 ^a^	0.001
PDW (%)	51.9 ± 24.4 ^a^	37.6 ± 23.7 ^b^	27.5 ± 21.5 ^ab^	22.9 ± 8.4 ^c^	24.7 ±10.03 ^ab^	34.4 ±17.01 ^ab^	0.001

^a,b,c^ Superscript letters show significant difference (*p* ≤ 0.05). ^1^ Dietary treatments where C = control (without vitamin B12), all other are vitamin B12 supplemented groups. Each mean represents values from 8 replicates (2 ducks/replicate).
